# Comparison of Different Methods for Estimating Cardiac Timings: A Comprehensive Multimodal Echocardiography Investigation

**DOI:** 10.3389/fphys.2019.01057

**Published:** 2019-08-22

**Authors:** Parastoo Dehkordi, Farzad Khosrow-Khavar, Marco Di Rienzo, Omer T. Inan, Samuel E. Schmidt, Andrew P. Blaber, Kasper Sørensen, Johannes J. Struijk, Vahid Zakeri, Prospero Lombardi, Md. Mobashir H. Shandhi, Mojtaba Borairi, John M. Zanetti, Kouhyar Tavakolian

**Affiliations:** ^1^Electrical and Computer Engineering Department, University of British Columbia, Vancouver, BC, Canada; ^2^Heart Force Medical Inc., Vancouver, BC, Canada; ^3^IRCCS Fondazione Don Carlo Gnocchi, Milan, Italy; ^4^School of Electrical and Computer Engineering, Georgia Institute of Technology, Atlanta, GA, United States; ^5^Department of Health Science and Technology, Aalborg University, Aalborg, Denmark; ^6^Department of Biomedical Physiology and Kinesiology, Simon Fraser University, Burnaby, BC, Canada; ^7^Fraser Health Authorities, Burnaby, BC, Canada; ^8^Acceleron Medical Systems, Arkansaw, WI, United States; ^9^Electrical Engineering Department, University of North Dakota, Grand Forks, ND, United States

**Keywords:** cardiac time intervals, phonocardiography (PCG), impedance cardiography (ICG), seismocardiography (SCG), echocardiography, pre-ejection period (PEP), left ventricular ejection time (LVET)

## Abstract

Cardiac time intervals are important hemodynamic indices and provide information about left ventricular performance. Phonocardiography (PCG), impedance cardiography (ICG), and recently, seismocardiography (SCG) have been unobtrusive methods of choice for detection of cardiac time intervals and have potentials to be integrated into wearable devices. The main purpose of this study was to investigate the accuracy and precision of beat-to-beat extraction of cardiac timings from the PCG, ICG and SCG recordings in comparison to multimodal echocardiography (Doppler, TDI, and M-mode) as the gold clinical standard. Recordings were obtained from 86 healthy adults and in total 2,120 cardiac cycles were analyzed. For estimation of the pre-ejection period (PEP), 43% of ICG annotations fell in the corresponding echocardiography ranges while this was 86% for SCG. For estimation of the total systolic time (TST), these numbers were 43, 80, and 90% for ICG, PCG, and SCG, respectively. In summary, SCG and PCG signals provided an acceptable accuracy and precision in estimating cardiac timings, as compared to ICG.

## 1. Introduction

Cardiac time intervals have clinical significance in mitral valve stenosis, coronary artery disease (Boudoulas, 1990; Reant et al., 2010), arterial hypertension (Brubakk et al., 1987), atrial fibrillation, hypovolemia and fluid responsiveness (Tavakolian et al., 2014), chronic myocardial disease (Reant et al., 2010) and in the assessment of left ventricular performance (Boudoulas, 1990; Reant et al., 2010). These intervals present a temporal description of the sequential phases of a cardiac cycle. Some of the important cardiac intervals include pre-ejection period (PEP), defined as the time period between the onset of left ventricular depolarization (the onset of QRS complex on electrocardiogram (ECG), and in particular the ECG Q wave when available) and the opening of the aortic valve (Umar and Leyva, 2012); left ventricular ejection time (LVET), defined as the interval between aortic valve opening and closure events; total systolic time (TST), defined as the time between ECG Q and the closure of the aortic valve; and electromechanical delay (EMD), defined as the time interval between ECG Q and the closure of the mitral valve (Que et al., 2002; Badano et al., 2007). Estimation of cardiac intervals involves detecting the timing of the opening and closure of the aortic and mitral valves.

**Figure 1:**
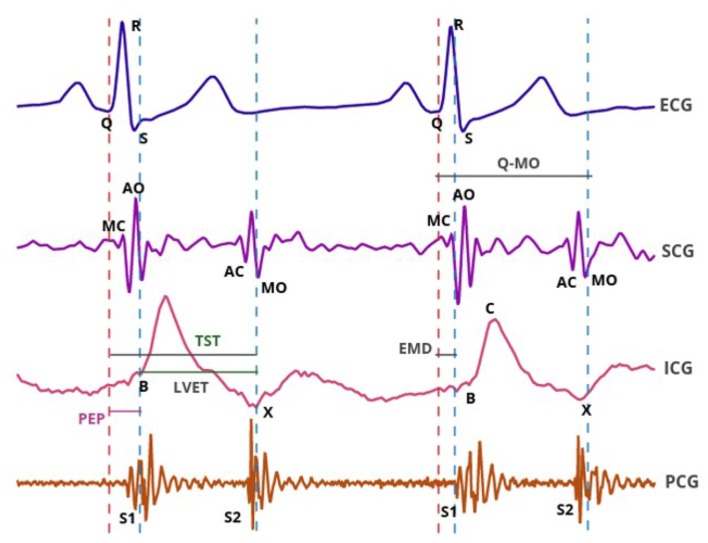
Simultaneous sample recordings of ECG, SCG, ICG, and PCG signals captured from a 40-year-old male participant in the supine position. The SCG MC and SCG MO points correspond to mitral valve closure and opening; the SCG AC and SCG AO points corresponded to the aortic valve closure and opening. ICG B point corresponded to aortic valve opening and ICG X point to aortic valve closure. EMD, PEP, TST and LVET systolic time intervals are also illustrated. S1 and S2 waves on PCG corresponded to mitral and aortic valve closure, respectively.

In clinical settings, the opening and closure of the aortic and mitral valves are commonly measured noninvasively using different ultrasound modalities such as M-mode, Doppler flow imaging, Tissue Doppler Imaging (TDI) or speckle tracking strains. These methods are time-consuming and require a trained sonographer to obtain accurate cardiac images. As such, there is a growing interest in the search for alternative simpler techniques to measure cardiac intervals. Phonocardiography (PCG), impedance cardiography (ICG) and seismocardiography (SCG) have been extensively used for this purpose (Figure 1). The non-invasive nature of these technologies makes them well-suited for inclusion in wearable solutions (Di Rienzo et al., 2013, 2014; Ruiz et al., 2013; Chen et al., 2015). This paper provides a unique and comprehensive analysis of the accuracy of cardiac timings estimated using PCG, ICG and SCG recordings, as compared to standard echocardiography methods.

### 1.1. Background

PCG is the measure of the heart sounds and is captured using a stethoscope and microphone. These sounds are generated by valve closure as well as by blood flow turbulence during systole and diastole. In a normal heart, two dominant sounds, S1 and S2, appear in rhythmical form (Granados et al., 2015). S1, the first heart sound, occurs when the mitral valve closes (the start of systole). S2, the second heart sound, occurs at the end of systole and is related to the closure of the aortic valve. By determining the beginning of S1 and S2 on the PCG signals, and the onset of QRS wave on ECG, EMD and TST intervals can be estimated. Since PCG signals do not contain information related to the opening of the aortic valve, PEP cannot be extracted from the PCG recordings (see Figure 1).

ICG is a technology which measures the thoracic impedance. During each cardiac cycle, the change in blood volume of the thoracic arterial system, results in a change in the electrical conductivity and the impedance of the thorax. The impedance changes are primarily due to changes in the velocity and volume of the blood in the aorta (Bernstein and Lemmens, 2005; Henry et al., 2011). The fiducial points on the first derivative of an impedance waveform (B and X), have been proposed to coincide with aortic valve opening and closure, respectively, making it possible to measure LVET, which was subsequently used to estimate stroke volume and other hemodynamic parameters (see Figure 1). In addition, there have been subsequent efforts to use ICG independently to approximate PEP (Sherwood et al., 1990; Burlingame et al., 2013).

SCG captures the chest acceleration induced by the motion of myocardium recorded using an accelerometer commonly mounted on the lower part of the sternum. In 1957, SCG was recorded under the name of precordial ballistocardiogram (Mounsey, 1957) and was used in the early 1960s for monitoring heart rate variability (Baevskii et al., 1964). Afterward, in the late 1980s, SCG was introduced as a technology for monitoring cardiac function (Salerno and Zanetti, 1991). In a study conducted by Crow et al. (1994), the fiducial points of SCG, labeled as MC, AO, AC, and MO were found to correspond to mitral valve closure, aortic valve opening, aortic valve closure and mitral valve opening, respectively, and validated against the echocardiography images (Crow et al., 1994). Recently, Sørensen et al. (2018) conducted a study to define fiducial points in the SCG recordings obtained from forty-five healthy individuals. In each subject the SCG waveforms were averaged and the points were then correlated with the cardiac events identified in ultrasound images.

The main purpose of our study was to provide a comprehensive validation of the accuracy of cardiac intervals estimated using PCG, ICG and SCG, as compared to the measurements made using echocardiography. An international team of researchers with expertise in non-invasive cardio-mechanical signals annotated the fiducial points on PCG, ICG and SCG recordings and estimated the cardiac intervals with respect to ECG Q. Later, we compared these estimates with the echocardiographic measures of the same cycles.

This study extended previous studies with the following aspects: (1) the simultaneous recording of PCG, ICG, and SCG which made it possible to compare the cardiac interval estimates from three different methods; (2) the recruitment of a larger number of participants (eighty-six individuals); (3) beat-to-beat annotation of fiducial points without ensemble averaging over cardiac cycle. Averaging may remove the beat to beat variations and could introduce errors due to changes in heart rate; as such, every individual cardiac cycle was annotated separately leading to the analysis of more than two thousand separate cardiac cycles; (4) the use of multimodal echocardiography, M-mode, Doppler and TDI; (5) suggesting a new method to measure the heart valve opening and closure using electrocardiography. While echocardiography is commonly used in clinical cardiology for annotating the timing of the cardiac valve opening and closure and measuring cardiac intervals, it has its own imprecisions mostly induced by the intrinsic noise of the images and the lack of agreement between the measurements of different sonographers. To address this issue we suggested a new and different measurement protocol introducing valve opening and closing time ranges. Rather than associating each valve movement with a single time instant, the timing of the valve opening or closure event was associated with a time window ranging from the initiation to the completion of the event; and (6) The annotations for each different technology of ICG, PCG, and SCG were performed by experts in each field and not a single group.

## 2. Materials and Methods

### 2.1. Participants

Eighty five healthy, male and female (*n* = 51) adults between 19 and 85 years of age (age: 27.8 ± 10.3, BMI: 24.2 ± 5.00) were recruited for this study. Subjects with known history of cardiovascular, respiratory, or major musculoskeletal injuries were excluded from recording. The participants were initially scanned with echocardiography to detect any visible cardiac anomalies including valvular regurgitations and pre-existing congenital heart disease.

This study was carried out in accordance with the recommendations of Simon Fraser University policies and procedures involving human participants with written informed consent from all subjects. All subjects gave written informed consent in accordance with the Declaration of Helsinki. The protocol was approved by the Office of Research Ethics at Simon Fraser University, Vancouver, Canada.

### 2.2. Data Acquisition

Two pairs of ICG sensors were placed on the neck and on the mid-axillary line at the xiphoid process level to measure the ICG signals (BoMed Inc., NCCOM3, USA). A low-noise 3-axial MEMS joint accelerometer-gyroscope sensor (ASC GmbH, ASC IMU 7.002LN.0750, Germany) was used to record SCG. The sensor was mounted on the sternum close to the xiphoid process and secured by double-sided tape. The PCG signals were recorded using a digital stethoscope mounted on the middle of sternum (Thinklabs digital stethoscope, CO, USA). Simultaneously, a reference two-lead ECG (iWorx Systems, Inc., IX-BIO8-SA, NH, USA) was recorded. All recordings were conducted with iWorx data acquisition system (iWorx Systems, Inc., IX-416, NH, USA), sampled at 1,000 Hz with 16-bit resolution.

A Vivid q portable ultrasound machine (GE Medical Systems, New York, US) was used for recording echocardiograms. To synchronize between the iWorx data acquisition system and echocardiography machine, separate ECG signals were used as input to these machines and their electrodes were placed close to each other on the shoulders to create more similarity in ECG morphologies.

All data recordings were performed at the Aerospace Physiology Lab at Simon Fraser University, Canada.

### 2.3. Echocardiography Protocol

Echocardiography is a standard modality extensively used in clinical settings for a variety of diagnostic purposes. However, its accuracy in finding the exact instant of valve opening or closing, besides being affected by noise and operator variability, as mentioned in the background section, it is also limited by the resolution of the captured frame and its poor synchrony with the ECG (Noda et al., 2017). To overcome these imprecisions, we proposed a new protocol for recording and labeling the echocardiogram images by performing a multimodal echocardiographic procedure and considering time windows for the assessment of the valve openings and closures. To avoid artifacts in the signals subjects underwent an echocardiographic scan in the supine position. If necessary, participants were only slightly tilted to the left lateral position to improve the quality of echocardiography.

#### 2.3.1. Multimodal Echocardiography

##### 2.3.1.1. M-mode

**Figure 2:**
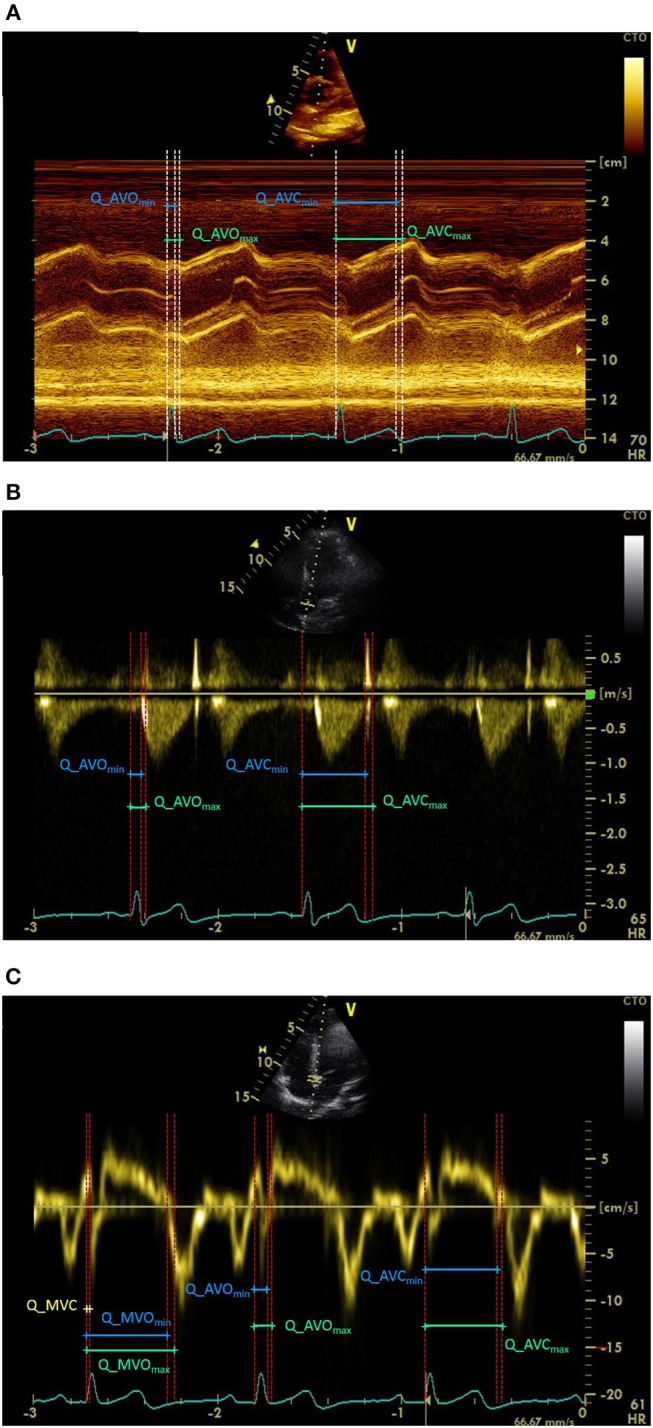
Echocardiogram images captured using **(A)** M-mode, **(B)** Doppler flow, and **(C)** TDI modalities. AVO and AVC stand for aortic valve opening and closure. MVO and MVC stand for mitral valve opening and closure. Max and min subscripts represent the start and the end of the echocardiographic ranges.

M-mode was used to demonstrate the excursion of the aortic valve cusps (Figure 2A). To improve the quality of images, special attention was taken to choose the angle through which the M-mode cursor was placed on the valve at a specific plane of cut. The M-mode images were not sufficiently accurate for measuring mitral valve opening and closure due to ambiguities produced by highly vibrating thin floppy leaflets attached to chordal apparatus.

For M-mode, in many cases the ascending aorta showed a steep upward motion in systole and returned to its original position in diastole taking the cusps out of the focused region. This made it impossible to detect both the aortic valve opening and closure in the same cardiac cycle (second cycle in Figure 2A). For these cycles, only the aortic valve opening, or closure was labeled.

##### 2.3.1.2. Doppler Flow

Doppler Flow was used to acquire spectral flow Doppler of blood across the aortic valve in apical-5 or apical-3 chamber views, whichever was more parallel to the flow across the valve (Figure 2B). To optimize the spectral Doppler flow images for measuring the timing of aortic events, special attention was given to collect the sample flow from the center of the flow jet through the central proximal region of the ascending aorta. To avoid attenuation of the images by lung tissues in the supine position, for some participants the Doppler flow images were acquired at the end of exhalation.

##### 2.3.1.3. TDI

TDI was used to measure myocardium velocity during each cardiac cycle by placing the sample volume in the ventricular myocardium immediately adjacent to the mitral annulus in apical four-chamber view. This relatively new modality of the echocardiogram technique allowed the time intervals of the aortic and mitral valves to be measured with increased consistency (Figure 2C).

#### 2.3.2. Valve Opening and Closure Ranges

Since the valve opening and closure occurs over a period from the initiation of the event until the completion point, rather than reporting a single time, we reported a time range for each event. As such, for the aortic valve, using the M-mode modality, the initial time of opening and the full opening of the cusps were labeled as AVO_*min*_ and AVO_*max*_, respectively (Figure 2A). The same for aortic valve closure, AVC_*min*_ was marked as the initiation of the closing aortic cusps and AVC_*max*_ was marked at the exact instant after the complete closure of the cusps.

On the Doppler flow images, the AVO_*min*_ was labeled as the moment before the blood flow (no-flow-yet point); the AVO_*max*_ was annotated as the moment when the blood flow was observed. AVC_*min*_ was marked as the point before the blood flow stopped and AVC_*max*_ was marked at the point after that there was no blood flowing (Figure 2B).

On TDI images, the AVO_*min*_ was marked at the exact moment before the annulus descended toward the apex and the AVO_*max*_ was labeled as the point when the annulus started descending toward the apex. At the end of systole, the myocardium reaches negative velocity. As the open aortic valve suddenly closes, there is a slight bounce, resulting in a shift from negative to positive velocity; AVC_*min*_ and AVC_*max*_ were marked, respectively, as the exact moments before and after the bounce (Figure 2C). As well, the exact moment before the annulus ascended away from the apex (when the myocardium velocity shifted from positive to negative) was labeled as MVO_*min*_. The exact moment after the annulus had started ascending away from the apex was labeled as MVO_*max*_ (Figure 2C). MVC was always annotated as a single point annotation, rather than a range like the other points (Figure 2C).

### 2.4. Manual Annotations of ICG, SCG, and PCG

Cardiac timings are traditionally defined relative to the onset of ECG QRS complex, which is considered as the starting point of cardiac contraction. However, for some subjects the start of QRS (Q-wave) was not easy to be detected. In these cases, we started with the neighboring R-wave and used the valley immediately located before the Q-wave.

Estimation of cardiac intervals from the ICG signal required annotation of the characteristic points of B and X, which are assumed to coincide with the opening and closing of the aortic valve. In this study, ICG B was annotated as the local minimum on the notch to the left of point C (Figure 1) and the X point was annotated as the time instant where the lowest ICG value occurred after point C (Carvalho et al., 2011). PEP_*icg*_ was measured as the interval from ECG Q to ICG B and TST_*icg*_ was obtained as the interval from ECG Q to ICG X. LVET was calculated as the timing interval between B and X points.

On PCG signals, the S1 and S2 sounds were annotated by the expert; EMD_*pcg*_ and TST_*pcg*_ were estimated as the interval from the ECG Q to the beginning of S1 and S2, respectively. The onset of the S1 sound was defined as the onset of the first peak after the ECG Q which had a height that exceeds the max amplitude of the preceding diastolic period. The onset of the S2 sound was defined as the onset of the first sharp negative wave in the S2 sound.

It was proposed that the SCG fiducial points of MC, MO, AC, and AO would coincide with mitral valve and aortic valve closing and opening, respectively. For the annotation of these points the traditional nomenclature proposed by Crow et al was considered (Crow et al., 1994). On this basis, PEP_*scg*_ was obtained from ECG Q to the SCG AO point and TST_*scg*_ was measured from ECG Q to the SCG AC point (Figure 1). EMD_*scg*_ and Q-MO_*scg*_ were measured as the intervals from ECG Q to the SCG MC and MO points, respectively. LVET_*scg*_ was measured as the time interval between SCG AO and SCG AC.

### 2.5. Statistical Analysis

Accuracy was assessed as the difference of the SCG, PCG and ICG measurements with respect to echocardiography measurements. All the results were presented for every individual modality of echocardiography (M-mode, Doppler and TDI) and also for all cycles from all modalities together.

The percentage of cycles where the ICG, PCG, or SCG annotated fiducial points fell inside the, 5-ms margins of, corresponding echocardiography ranges were reported. The choice of a 5-ms margin was due to the time resolution limitations of the GE Vivid q system.

PEP estimation error (errpep) were calculated using the following equation:

(1) $$\begin{array}{r} err_{pep}=100*\frac{abs\left(PEP_{ref}-PEP_{est}\right)}{PEP_{ref}} \end{array}$$

where PEP_*est*_ represents PEP_*icg*_ or PEP_*scg*_ and PEP_*ref*_ represents PEP_*mmode*_, PEP_*doppler*_ or PEP_*tdi*_. PEP_*ref*_ was measured from ECG Q to the middle point of AVO_*min*_ to AVO_*max*_. A similar formula to Equation (1), was used to calculate the estimation error for ST, EMD and Q-MO.

The agreement between ICG, PCG and SCG estimated time intervals and the reference echocardiogram intervals, using the middle-point of the echocardiography range, were assessed using the multiple-observation Bland-Altman method (Bland and Altman, 1986). Bias, 95% limits of agreement (LOA) and two standard deviations (2SD) were reported to quantify the distributions of error.

In addition, the interclass correlation coefficient (ICC) was estimated as a reliability index. ICC reflects both the degree of correlation and the agreement between measurements. ICC was estimated as a ratio of reference variance over reference variance plus error variance. Based on the 95% confidence interval of the ICC estimate, values less than 0.5, between 0.5 and 0.75, between 0.75 and 0.9, and greater than 0.90 are indicative of poor, moderate, good, and excellent reliability, respectively (Koo and Li, 2016).

Since the annotation of SCG, ICG and PCG fiducial points were manually performed by the different annotators, a separate independent annotator was trained to annotate all the same recordings of SCG, ICG and PCG. This was used to provide a quantification of annotator variability and evaluate the ease of annotation of fiducial points for every modality. ICC was estimated to show the agreement between annotators.

## 3. Results

### 3.1. PEP Measurements

M-mode was available for 85 participants, with a total of 504 measurements of AVO_*min*_ and AVO_*max*_. Doppler flow was available for 59 participants giving a total of 292 cardiac cycles and TDI was available for 53 participants giving a total of 256 cardiac cycles. The average interval between AVO_*min*_ and AVO_*max*_ was estimated to be 19.3 ms, 16.4 ms and 14.7 ms for M-mode, Doppler and TDI, respectively.

The results for all PEP measurements are listed in Table 1. For 55% of cardiac cycles, ICG B points fell within M-mode AVO_*min*_ and AVO_*max*_. Bias and 2SD between PEP_*icg*_ and PEP_*mmode*_ (the mid-point of echo range) were estimated as 11.7 ms and 29.24 ms, respectively. The average of error was estimated as 23.2% using Equation 1. For 83% of the cycles, the SCG AO fell within the M-mode AVO_*min*_ and AVO_*max*_ range. The agreement between PEP_*scg*_ and PEP_*mmode*_ was assessed by the Bland-Altman method with a bias of 2.1 ms and 2SD of 26.0 ms. The average of error was estimated as 12.5%.

**Table 1:** Estimated cardiac time intervals compared to the ones measured using the different modalities of echocardiography.

**Cardiac timing**	**Echocardiogram modality**	**2SD (ms)**	**Bias (ms)**	**ICC**	**Error%**	**(%) in Range**
PEP_*scg*_	M-mode	26.0	2.1	0.74	12.5 ± 12.0	83
	Doppler	21.5	0.5	0.83	10.9 ± 14.8	90
	TDI	23.2	3.7	0.82	13.1 ± 18.1	86
	All	24.7	2.5	0.59	12.8 ± 16.5	86
PEP_*icg*_	M-mode	29.2	11.7	0.34	23.2 ± 17.4	55
	Doppler	28.7	12.5	0.46	22.9 ± 20.8	54
	TDI	31.9	15.7	0.60	29.0 ± 22.8	37
	All	30.1	13.5	0.35	25.5 ± 20.6	47
TST_*scg*_	M-mode	13.6	−0.2	0.97	1.4 ± 1.1	92
	Doppler	14.8	7.6	0.91	2.4 ± 1.6	83
	TDI	15.7	−0.5	0.96	1.6 ± 1.3	92
	All	16.2	2.0	0.97	1.4 ± 3.2	90
TST_*icg*_	M-mode	56.0	15.4	0.84	5.2 ± 6.3	52
	Doppler	66.0	25.6	0.65	8.0 ± 7.6	30
	TDI	51.6	13.5	0.83	4.9 ± 6.1	40
	All	55.8	17.8	0.61	6.0 ± 7.0	43
TST_*pcg*_	M-mode	21.5	−3.4	0.93	2.0 ± 1.9	80
	Doppler	18.0	5.1	0.86	2.3 ± 1.6	82
	TDI	19.6	−3.3	0.95	2.1 ± 1.8	78
	All	21.8	−0.8	0.94	2.1 ± 1.8	80

*The last column shows the percentage of estimated cardiac timings dropping in the 5-ms margins of corresponding echocardiography range*.

**Figure 3:**
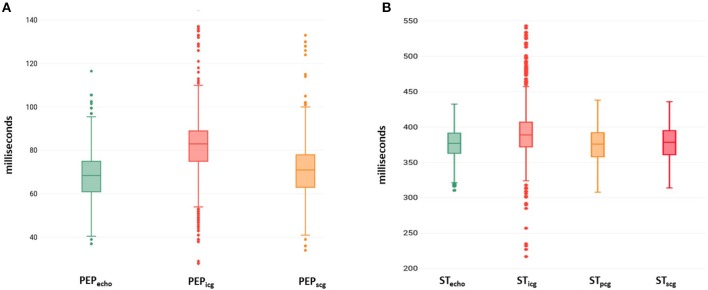
The boxplot shows the values of **(A)** PEP estimated from ICG and SCG and **(B)** TST estimated from ICG, PCG, and SCG compared to the measurements from echocardiography. Lower quartile, median, and upper quartile values were displayed as bottom, middle, and top horizontal line of the boxes. Whiskers were used to represent the most extreme values within 1.5 times the interquartile range from the quartile. Outliers (data with values beyond the ends of the whiskers) were displayed as dots.

**Figure 4:**
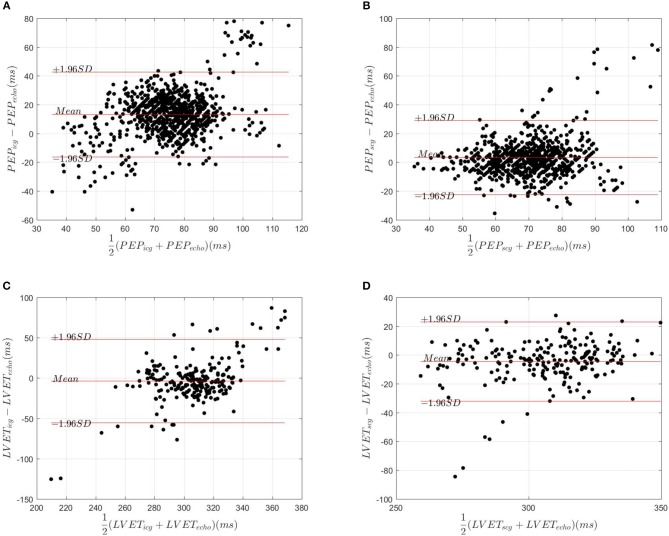
Bland and Altman plots for assessing the agreement between **(A)** PEP_*echo*_ and PEP_*icg*_, **(B)** PEP_*echo*_ and PEP_*scg*_, **(C)** LVET_*echo*_ and LVET_*icg*_, and **(D)** LVET_*echo*_ and LVET_*scg*_.

The value of PEP_*echo*_, PEP_*icg*_ and PEP_*scg*_ for all cycles were compared in Figure 3A. For all echocardiography measurements of AVO_*min*_ and AVO_*max*_ from M-mode, Doppler and TDI modalities, 47% of ICG B and 86% of SCG AO occurred in their corresponding echocardiography ranges. The average percentage error between PEP_*echo*_ and PEP_*icg*_ and PEP_*scg*_ were estimated at 25.5% and 12.8%, respectively. The agreements between PEP_*echo*_ and PEP_*scg*_ and PEP_*icg*_ were assessed by the Bland-Altman plot (Figure 4).

### 3.2. TST Measurements

The comparison between TST_*icg*_, TST_*pcg*_, and TST_*scg*_ estimates and the corresponding measurements from different echocardiography modalities are presented in Table 1. TST was the only timing parameter that could be estimated by all three technologies (ICG, PCG, and SCG). The values of TST_*echo*_, TST_*icg*_, TST_*scg*_, and TST_*pcg*_ for all cycles are depicted in Figure 3B. For all cardiac cycles, 43%, 90%, and 80% of annotated corresponding aortic valve closure points on ICG, PCG, and SCG signals fell within the AVC echocardiography range, respectively. The value of 2SD between TST_*echo*_ and TST_*icg*_, TST_*pcg*_, and TST_*scg*_ were calculated as 55.8, 21.8, and 16.3 ms, respectively. ICC between TST_*echo*_ and TST_*icg*_, TST_*pcg*_, and TST_*scg*_ were estimated as 0.61, 0.94, and 0.97, respectively.

### 3.3. EMD and Q-MO Measures

TDI images were used for measuring the timing of mitral valve opening and closure. The average EMD, was calculated over 211 cardiac cycles, was 31.5 ± 7ms using TDI echocardiography, 36.8 ± 9.7 ms using PCG, and 31.9 ± 9.8 ms using SCG. For 46% of all cycles, PSG S1 fell in the 5 ms vicinity of MVC_*tdi*_ while this value was 45% for MC_*scg*_. The average of error between EMD_*scg*_ and EMD_*pcg*_ with EMD_*tdi*_ were 24.0% and 28.5%, respectively.

For 44% of all the measurements, MO_*scg*_ fell within the MVO_*min*_ and MVO_*max*_ interval. The agreement between MO_*scg*_ and MVOtdi was assessed by a Bland-Altman analysis with the bias of -19.00 ms and the 2SD of 27.6 ms (Table 2).

**Table 2:** The comparison of estimated EMD_*pcg*_ and EMD_*scg*_ compared to reference EMD_*echo*_ estimated using the TDI echocardiography and comparison of the SCG Q-MO intervals to the same intervals estimated from TDI images.

	**2SD (ms)**	**Bias (ms)**	**In ranges (%)**	**Percentage errors**	**ICC**
EMD_*pcg*_	18.0	4.3	46	28.5 ± 29.9	0.59
EMD_*scg*_	14.19	0.07	45	24.0 ± 20.2	0.45
Q-MO_*scg*_	27.62	−19.00	44	4.6 ± 2.7	0.68

### 3.4. LVET Measurements

For LVET measurements, we only used the TDI method which could provide reliable simultaneous measurement of aortic valve opening and closure for 237 cardiac cycles. The measurement of LVET was also provided from ICG and SCG by finding the B to X interval for ICG and AO to AC interval for SCG. 2SD between LVET_*tdi*_ and LVET_*icg*_ and LVET_*scg*_ were estimated at 52.8 ms and 28.1 ms, respectively. The biases were around 4 ms for both SCG and ICG. The percentage errors were 5.7% and 3.2%, and ICC was 0.60 and 0.81, for ICG and SCG, respectively.

### 3.5. Between Annotator Variability

To measure the reliability between annotators, an independent person was trained to annotate all fiducial points of ICG, PCG and SCG recordings. This annotator was blind to the results of echocardiography and other annotators. ICC was estimated to measure the agreement between annotators. For ICG PEP and TST, the ICC values between annotators were 0.45 and 0.76, respectively. The PEP and TST obtained from SCG, the ICC between two annotators were 0.78 and 0.93. For PCG TST, the ICC values was 0.93.

### 3.6. Gender Variability

Two evaluate the agreement between SCG, PCG and ICG estimates and echo measurements in two groups of males and females, the PEP and TST intervals were estimated separately for men and women. 2SD between PEP_*scg*_ and PEP_*echo*_ were estimated as 17.5 ms and 17 ms for men and women, respectively; the percentage of error between PEP_*scg*_ and PEP_*echo*_ were estimated as 12.7 ± 6.7 and 12.0 ± 6.8 for males and females. 2SD between TST_*scg*_ and TST_*echo*_ were estimated as 16.2 ms and 16.4 ms for men and women and the percentage of error between TST_*scg*_ and TST_*echo*_ were estimated as 5.9 ± 0.8 and 5.8 ± 0.8, respectively. These results suggested that there were no significant differences regarding the agreement between SCG estimates and echo measurements in men and females. The similar results were observed for PCG and ICG estimates in compare to echo measurements for two groups of males and females.

## 4. Discussion

The primary focus of this study was to investigate and quantify the reliability of the available non-invasive methodologies with the potential to be embedded in wearable devices (ICG, SCG, and PCG), for detecting the clinically relevant cardiac timings. The results of this study can be used for informing future development of wearable devices for ubiquitous assessment of cardiac timing intervals. These results suggest that acoustic (PCG) and vibration (SCG) signals are more precise and accurate, compared to ICG, for such applications. The findings of this study can be summarized as follow:

1. The PEP derived from ICG significantly deviates from the echocardiography range (for more than 50% of cycles) while the same parameter extracted from SCG was in the echocardiogram range for 86% of cycles.
2. TST_*pcg*_ and TST_*scg*_ estimation error were less than TST_*icg*_ estimation error; however, even for estimating TST_*icg*_, these errors were small (about 6%), which could be negligible in some applications.
3. LVET estimation error was negligible for both ICG and SCG recording. However, the distribution of error (2SD) was almost twice as high for ICG.
4. EMD estimated from PCG and SCG recording delivered a very high average error accounting to about a quarter of the value of the interval itself. This could be totally in the margin of error of the GE Vivid q echocardiography device, and probably most other clinically available devices, making it difficult to draw a significant conclusion on this timing.
5. The variability between annotators was most significant in annotating the fiducial points on the ICG recordings.

The annotations of ICG, PCG and SCG were undertaken by an international group of experts. These timings were compared with the same timings measured using three different echocardiography methods, currently used in the clinical practice (M-mode, Doppler, and TDI). Rather than an absolute single assignment for valve opening or closure, echocardiography time ranges were compared with. No ensemble averaging was used and individual annotation of more than 2,120 cardiac cycles was undertaken. Ensemble averaging could smooth the outliers and possibly improve the results. However, in a real clinical setting where the measurements are only available for a few cycles, if not a single cycle, ensemble averaging is not possible. Moreover, the variation of heart rate could affect the performance of ensemble averaging.

### 4.1. ICG

In only 47% of cycles, ICG B occurred in the echocardiography AVO range and PEP_*icg*_, on average, differed by about 25% from PEP_*echo*_. Compared to PEP estimation, LVET_*icg*_ and TST_*icg*_ both provided better estimates for LVET and TST with only 5.7% and 6% deviation from the echocardiography measurements. The initial claim for correspondence of the ICG B point to aortic valve opening was made by Sherwood et al. (1990) which rooted in the two studies from the 1980s. The first study (Petrovick et al., 1980) included only a single plot of M-mode and ICG together and did not have any quantitative comparison between the measurements. The second study (Stern et al., 1985), only provided results for LVET and indicated that ICG estimates over-estimates the echo measurement. Later, the LVET estimated from ICG was used in the formula for estimation of stroke volume (Sherwood et al., 1990). This might have created a misunderstanding, from early on, that PEP also could be estimated from ICG recordings independently and accurately. Neither our current results nor any results from previous studies—of which we are aware—quantitatively prove this supposition. There is a recent effort reporting similar results, as in current study, when it comes to ICG (Carvalho et al., 2010).

### 4.2. PCG

The TST interval was measured using PCG with a negligible average error of 2.1%. However, the EMD estimation error was 24%. There was also a bias of 4.3 ms between EMD_*pcg*_ and EMD_*tdi*_, showing that EMD_*pcg*_ has been overestimated by 5.1 ms on average. Considering the short duration of EMD this contributed to a significant error in the estimation of mitral valve closure.

### 4.3. SCG

For 86% of the cycles, SCG AO took place in the echocardiography AVO range. For PEP_*scg*_ estimation, the average error was about 12.8%. For ST_*scg*_ estimation error was 1.4% on average. The annotated AC point on the SCG recordings provided very close estimates to the echocardiography AVC measures. These results were in line with a recent study on the SCG signal (Sørensen et al., 2018). For EMD_*scg*_ the average error was about 21.7%, almost the same as EMD_*pcg*_.

The Q-MO had an average difference of 4.6% with echo Q-MO and on average occurred 19 ms behind the echo measurement. This bias was expected and matched with the original research, conducted by Salerno (1990), which compared echocardiogram with SCG and reported the worst diastolic timing at MO.

### 4.4. Different Echocardiography Methods

From Tables 1, 2 no significant difference between ICG, SCG and PCG is observed when it comes to the modality of the echocardiography. The same relation holds using either M-mode, Doppler or TDI.

### 4.5. Between Annotator Variability

The ICC was the highest for TST extraction using both PCG and SCG, indicating the ease of training someone to annotate the point. The PEP extraction was challenging; this can be seen in lower ICC values for both ICG and SCG. The EMD values had ICC of 0.78, which is a higher value with respect to PEP.

The estimation of cardiac time intervals using PCG, SCG and ICG, investigated in this study, offers an opportunity to assess cardiac contractility which, in addition to the analysis of the other features of these signals, broadens the potential of SCG, PCG and ICG in the monitoring of cardiovascular performance. As these technologies yield themselves to wearable applications, they could be used outside of the hospital/clinical settings to detect the potential abnormalities and malfunctions of the cardiovascular system such as heart failure (Inan et al., 2018), hypovolemia (Tavakolian et al., 2014), and hypotension (Brubakk et al., 1987) or to validate cardiac resynchronization therapy (Marcus et al., 2007). Moreover, this technology can be used to monitor the improvement of cardiac performance in healthy individuals as a result of the adoption of a healthier and more active lifestyle.

In this study, we strictly limited the subject population to healthy people and avoided any cardiac abnormalities. In addition, to avoid any effects of cardiovascular againg, i.e., arterial stiffness, rigid myocardium or valvular calcification, on the morphology of ICG, PCG and SCG signals, the individuals participated were very constrained to young people (age = 27.8 ± 10.3). Thus, the next step for this study would be to include older subjects and also subjects with various cardiovascular diseases to evaluate the methods further and to develop robust methodologies for fiducial point detection in such populations. It should also be noticed that in this study the signals were manually annotated. Automatic annotation of ICG, PCG or SCG recordings has its own challenges addressed in several studies. It should also be noted that in all the annotations the simultaneous ECG signal was considered as the reference of the annotations.

It should also be noticed that in this study the signals were manually annotated. Automatic annotation of ICG, PCG or SCG recordings has its own challenges addressed in several studies. It should also be noted that in all the annotations the simultaneous ECG signal was considered as the reference of the annotations.

The GE Vivid q device used in this study is regularly used in clinical environments and hospitals. However, there is an inherent dis-synchrony between recorded images and the ECG signal obtained in the most ultrasound devices. Assuming a similar error for ICG, SCG and PCG we do not believe such errors could change the relations of the obtained results with each other. However, a research grade ultrasound device could reduce such errors in future studies.

This study was limited to analysis of the z-axis of the accelerometer signal in the dorsoventral direction. The movement of the chest due to cardiac vibration is not limited to this direction; the manifests itself in the other two axes and also in rotational movements which can be picked up by Gyroscopes (Tadi et al., 2017). These additional signals were also recorded and, in the near future, we will analyze them to investigate the possibility of using all aspects of the vibrations to reduce the error between echocardiography and mechanical vibration annotations.

## Ethics Statement

This study was carried out in accordance with the recommendations of Simon Fraser University policies and procedures involving human participants with written informed consent from all subjects. All subjects gave written informed consent in accordance with the Declaration of Helsinki. The protocol was approved by the Office of Research Ethics at Simon Fraser University, Vancouver, Canada.

## Author Contributions

PD contributed to design the study, data acquisition and processed the obtained data, analyzed the results, prepared the figures, and revised the paper critically for content. FK-K contributed to the design of the study and revised the paper critically for content. AB revised the paper critically for content and helped with data acquisition. VZ assisted with SCG annotations and critically revising the report's content. MB was the certified sonographer who conducted echocardiography, annotated the echocardiogram. MD, PL, and PD annotated SCG cycles and read over and edited the manuscript. OI and MS annotated ICG cycles and read over and edited the manuscript. SS, JS, and KS annotated PCG cycles, read the paper, and provided feedback. JZ contributed to the design of the study. He passed away on November 29th, 2017. KT initiated the study, contributed to the design of the study, data acquisition and processed the obtained data, analyzed the results, and drafted the reviews.

### Conflict of Interest Statement

PD and VZ are employed by Heart Force Medical Inc., Vancouver, Canada. KT is on the Board of Directors at Heart Force Medical, Inc. Vancouver, Canada. FK-K is the CTO of Heart Force Medical Inc., Vancouver, Canada. Deceased JZ was employed by Acceleron Medical Systems, Wisconsin, USA. The remaining authors declare that the research was conducted in the absence of any commercial or financial relationships that could be construed as a potential conflict of interest.
